# Renal Trichodinosis in the Japanese Rice Fish, *Oryzias latipes* : First Report and Phylogenetic Classification

**DOI:** 10.1111/jfd.70212

**Published:** 2026-06-02

**Authors:** J. Harl, K. Lipnik, S. Thalguter, E. Lewisch

**Affiliations:** ^1^ Institute of Pathology University of Veterinary Medicine Vienna Austria; ^2^ Division of Fish Health University of Veterinary Medicine Vienna Austria

**Keywords:** 18S rRNA gene, Medaka, phylogenetics, *Trichodina* sp.

## Abstract

Investigation of a disease outbreak in a hobbyist's medaka ( *Oryzias latipes* ) collection led to the detection of *Trichodina* sp. in the kidney tubuli of individual fish. Based on this observation, a thorough clinical workup, including native microscopy, histopathology and molecular genetics, was conducted. Besides the trichodinids, *Ichtyobodo necator* and microsporidian gill infection were found in one out of the eight fish, and all of them were infected with *Mycobacterium* sp. Morphological description of the renal *Trichodina* sp. was based on the parasites found in renal tubules. For molecular genetic characterization and phylogenetic analysis, primers targeting the 18S rRNA gene of all members of the order Mobilida were designed. The phylogenetic analysis of the 18S rRNA sequences showed a close relationship to *Trichodina ruditapicis* (FJ499385), *Trichodina glossogobae* (ON042773) and *Trichodina sinonovaculae* (FJ499386) sampled from fish and mussels in China, differing by 2.2%–2.5%. Here we report for the first time renal trichodinosis of *O. latipes* and molecular data of a fish‐endozoic *Trichodina* sp.

## 1 Introduction

The Japanese rice fish, *Oryzias latipes* Temminck & Schlegel 1846, inhabits freshwater and brackish habitats in Eastern Asia. There are two distinct wild species in Japan, which differ in their geographical distribution, genetics and appearance. Several hundred varieties, differing in colour, body shape and fin shape, originate from these two species. Both the wild and the bred Japanese rice fish are known by the common name ’medaka’. In Japan, these fish have been kept as pets in small ponds and buckets since the 17th century (Sasado et al. 2010). Due to their small body size of less than 4 cm and their relatively low demands on husbandry, medakas also have become popular aquarium fish outside of Japan. Moreover, they serve as model organisms in a variety of research fields, including developmental biology, genetics, toxicology, environmental science, behavioural neuroscience, and as a model for human diseases (Walter and Obara 2015). The order Mobilida Kahl 1993 comprises up to five families, depending on the different authors (Lynn 2008). Among them, Trichodinidae Claus 1951 is the most species‐rich family. The number of genera included in this family is under ongoing revision, mostly based on molecular phylogenetics. While nine genera are listed following morphology‐based taxonomy by Van As and Basson (1989) and Lom and Dykova (1992), and ten and eleven genera by Basson and As (2006) and Lynn (2008), respectively, the World Register of Marine Species (WoRMS 2026) includes currently eight well established genera after synonymization and later revisions. All species of the order are peritrichous motile ciliates. Trichodinids can be found in both freshwater and marine habitats (Colorni 2008). They exhibit characteristic morphological features. The round, slightly flattened cell has an adhesive aboral disc, consisting of concentric skeletal denticles and radial pins (Lom 1973; Hausmann and Hausmann 1981a, 1981b). The distinct morphological features, such as the shape of the disc, the number and structure of the denticles, measurements of various structures and cell size, can be used to distinguish the species (Lom 1958; Wellborn 1967; de Oliveira Furtado et al. 2020). Still, morphological characteristics can vary within a single species, depending on the host, location and even season (Bradbury 1994; Kazubski 1980; Ogut and Altuntas 2011). Most *Trichodina* spp. are not strictly host specific (Lom and Dykova 1992) and a single host can be infested by more than one trichodinid species. Nevertheless, some species show a distinct host specifity pattern (Van As and Basson 1987). Although trichodinids mostly exhibit commensal or even symbiont lifestyles, feeding on bacteria and sloughed‐off cells of their hosts, they are commonly referred to as ectoparasites. This is most likely because in aquaculture and other artificial holdings, they can indeed cause irritation and pathology of skin and gills. Occasionally, trichodinids have been observed in the urinary tract of amphibians of the order Anura (Fulton 1923; Suzuki 1950; Kazubski 1980; Van Der Bank et al. 1989; Kruger et al. 1991; Collymore et al. 2013; Zhou et al. 2021). Some trichodinid species belonging to the genera *Trichodina* and *Paratrichodina* were reported from the urinary‐ or urogenital tract of fish (Table 1). Endoparasitic trichodinids often exhibit increased denticle number and well‐developed adhesive disc structures, which are suggested to be characteristic features of internal forms (Lom and Haldar 1976; Zhou et al. 2021). Nevertheless, these inferred observations do not permit assignment at the species level, nor do they allow differentiation between an exozoic and an endozoic lifestyle. On the other hand, molecular data of trichodinids are scarce, and for the endozoic species of fish they do not exist at all. Here we present the first observation of renal trichodinosis in medaka and the first molecular data of an endozoic fish‐trichodinid.

**TABLE 1:** Endogenus *Trichodina* spp. found in the genital‐ and urinary tract of fishes.

	FW/M	Host	Infestation site
*Vauchomia* ^a^ *renicola* Mueller 1938 syn. *Trichodina urinicola* Fulton 1923, ^a^	FW	*Esox niger*	Urinary bladder
*Vauchomia nephritica* Mueller 1938^a^	FW	*Esox masquinongy*	Urinary bladder, ureters, posterior third of kidney
*Trichodina urinaria* Dogiel 1941	FW	*Perca fluviatilis, P* *. flavescens* , *Stizostedion lucioperca*	Urinary bladder, ureters
*Trichodina oviducti* Poljanski 1955	M	*Raja* spp.	Urinary sinuses of both sexes; males: seminal groove, urogenital sinuses, and rectum; females: copulatory sac, rectum, oviducts
*Trichodina polycirra* Lom 1960	FW	*Rutilus rutilus, Abramis brama*	Urinary bladder, ureters
*Trichodina rajae* Evdokimova, Kuznetsova & Shtein 1969	M	*Raja scabina*	Oviducts
*Paratrichodina phoxini* Lom 1963	FW	*Phoxinus phoxinus*	Urinary bladder, ureters
*Paratrichodina alburni* Lom 1963	FW	*Alburnus alburnus*	Urinary bladder, ureters
*Trichodina schizothoraci* Aschurova & Stein 1972	FW	*Schizothorax intermedius*	Urinary bladder, ureters
*Paratrichodina degiustii* Lom & Haldar 1976	FW	*Notropis heterodon*	Urinary bladder, ureters
*Trichodina algonquinensis* (Li & Desser 1983)	FW	*Perca flavescens*, *Notropis cornutus*	Urinary bladder, ureters
*Trichodina uretra* Basson 1989	FW	*Barbus trimaculatus*	Urinary bladder, ureters
*Trichodina rhinobatae* (Van As & Basson 1996)	M	*Rhinobatos annulatus*	Urogenital tract
*Trichodina* sp. (Karlsbakk et al. 2002)	M	*Hydrolagus affinis*	Urinary system; males: also distal genital system
*Trichodina* sp. (Dang et al. 2019)	M	*Myxocephalus scorpius*	Blood vessels of spleen, kidney, liver (and others)

Abbreviations: FW, fresh water; M, marine.

^a^ The genus *Vauchomia* has been discarded and later reinstated at the genus level, which has led to the creation of synonyms.

## 2 Case Presentation, Methods and Results

Three medakas ( *Oryzias latipes* ) were submitted to the Fish Health Unit at the University of Veterinary Medicine, Vienna, in February 2025 by an aquarium hobbyist for investigation of ongoing low mortalities. Upon arrival, two of them showed swimming disturbances, and all of them were listless. One had a distended abdomen and showed forced respiration. The fish were euthanized using 500 mgL^−1^ buffered MS222. For microscopic observations, a light microscope (Olympus BX 53) was used. In the skin and the gill smear from one fish, *Ichthyobodo necator* (Kinetoplastida) was observed, and in the same fish, a few xenomas, presumably containing microsporidia, were detected in the secondary lamellae of the gills. No skin and gill parasites were found in the other fish. One fish was saved for in‐toto‐histology, whereas two were used for necropsy and further sampling. Both had abundant fat deposits, a pale liver, a large head kidney and a dark spleen (Figure 1). No granulomas were visible in liver, spleen, the kidney. While wet mounts of the liver and the spleen showed areas suspicious of necrosis, there were high numbers of *Trichodina* sp. in the collecting tubuli of the head and the trunk kidney of one fish. The parasites were documented using an Olympus digital camera DP72 and the software cellSense Standard (Olympus, Tokio, Japan) (Figure 2). Subsequently, kidney and visceral samples were aseptically removed from both fish for molecular examination and stored at −20°C until use. Additionally, Ziehl‐Neelson stains of organ imprints were prepared using routine protocols, allowing to detect high numbers of acid‐fast bacteria in the liver, spleen and kidney. To further investigate the presence of *Trichodina* sp. in kidney tubuli, five more medakas from the same aquarium were examined 10 days later. In none of them ectoparasites were present on the skin and gills, but *Trichodina* sp. was observed in the wet mount of an enlarged head kidney in one fish. Again, kidneys were sampled under sterile conditions and used for molecular examination.

**FIGURE 1:**
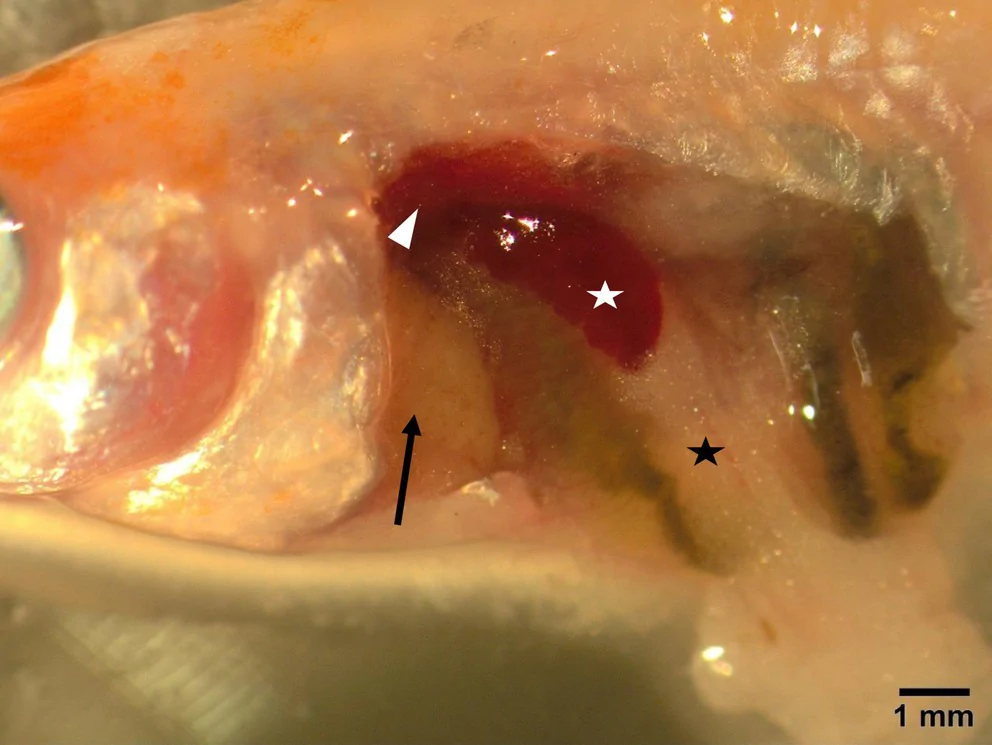
Situs of a medaka. Note the large head kidney (arrowhead), pale liver (arrow), spleen (white star), and abundant fat deposits (black star).

**FIGURE 2:**
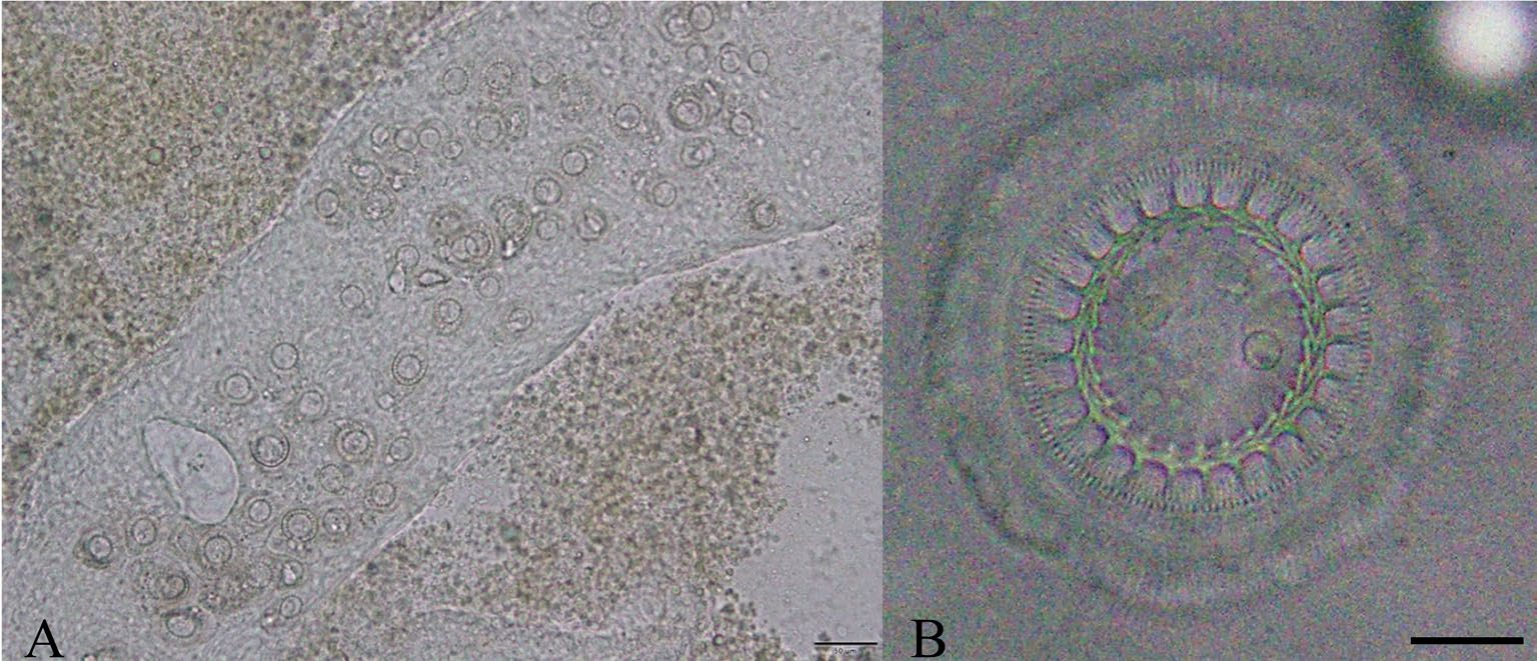
Light microscopy of a kidney smear of Medaka (A) numerous *Trichodina* sp. in kidney tubules (B) close up of a representative *Trichodina* sp. Scale bar = 10 μm.

DNA was extracted from kidney samples using the DNeasy Blood & Tissue Kit (QIAGEN, Venlo, Netherlands) according to the manufacturer's protocol. Two primer pairs were designed to amplify overlapping fragments of the 18S rRNA gene in members of the Mobilida, which together cover 1500 bp. The primers Mobilida_18S_F1 (5′‐AGT ATA AGT GCT RTA CGG CGA‐3′)/Mobilida_18S_R1 (5′‐ATC TGA TCG TCT TTG ATC CCC T‐3′) and Mobilida_18S_F2 (5′‐GTC GGG GGC ATT AGT ATT TAA TTG TC‐3′)/Mobilida_18S_R2 (5′‐CCA GAA GGT TCA CCA GAT CAC T‐3′) amplify 882‐bp and 780‐bp fragments of the 18S rRNA gene in all Mobilida, respectively. The alignment for designing the primers contained 760 complete or nearly complete 18S rRNA gene sequences from members of the Ciliophora, as well as numerous sequences from potential host species (fish and bivalves). The quality of the primers and the PCR profiles were checked using AmplifX v.2.1.1 (Nicolas Jullien, Aix‐Marseille Univ., CNRS, INP, Marseille, France; https://inp.univ‐amu.fr/en/amplifx‐manage‐test‐and‐design‐your‐primers‐for‐pcr).

The same conventional PCR protocol for reaction volumes of 20 μL was used for both primer pairs as follows: 2xDreamTaq Green PCR Master Mix (Thermo Fisher Scientific, Waltham, Massachusetts, USA), 10.0 μL; template DNA 1.0 μL; forward and reverse primer (10 μM), each 0.8 μL; and PCR grade water 7.4 μL. The initial denaturation was conducted at 95°C for 2 min, followed by 40 cycles of denaturation at 95°C for 30 s, annealing at 55°C for 30 s, elongation at 72°C for 1 min, and a final extension at 72°C for 5 min. *Trichodinella epizootica*, which was identified from a gill sample of a sturgeon, was used as a positive control.

PCR products were obtained from six out of the seven kidney samples and visualized on a 1.5% agarose gel (Serva Electrophoresis GmbH) stained with Gel Red (Biotium). Bands of the expected sizes were excised, purified with a MinEluteGel Extraction kit (Qiagen), and sequenced in both directions using the Sanger method at LGC Genomics (Berlin, Germany) with the corresponding PCR primers. The electropherograms of the sequences were visually inspected using BioEdit v.7.0.8.0 (Hall 1999). The sequences were uploaded to NCBI GenBank under the accession numbers PZ374349‐354.

The 18S rRNA gene sequences obtained from the six samples were identical. A BLAST search on NCBI GenBank showed the highest similarity to *Trichodina glossogobae* (ON042773), *Trichodina ruditapicis* (FJ499385), and *Trichodina sinonovaculae* (FJ499386) from fish in China, with 97.6%, 97.3% and 97.1% identity, respectively.

A phylogenetic tree was calculated with the 18S rRNA sequences of Mobilida and a sequence of *Euplotes minuta* (FJ876977) as outgroup. The sequences were aligned using MAFFT v7.3.1.1 (Katoh and Standley 2013) by applying the default settings. The alignment was trimmed to a length of 1138 positions, whereby the Mobilida sequences contained approximately 1160 bp (excluding gaps). The final alignment comprised 157 sequences, which were then collapsed to 66 haplotypes. A model test was performed with IQ‐TREE v. 3.0.1 (Nguyen et al. 2015), suggesting TIM2 + F + I + G4 as best‐fit substitution model according to the corrected Akaike Information Criterion. A Maximum Likelihood majority‐rule consensus tree with 1000 bootstrap replicates was calculated using IQ‐TREE v. 3.0.1. A Bayesian Inference tree was calculated with MrBayes v. 3.2 (Ronquist et al. 2012) by applying the next‐best available model (GTR + G + I), running the analysis for 5 million generations, and sampling every thousandth tree; after discarding the first 25% of trees as a burn‐in, a majority‐rule consensus tree was calculated from the remaining 3700 trees. The phylogenetic tree showed a division into two clades containing sequences of the genera *Trichodina* and *Urceolaria* (Figure 3). The *Trichodina* species analysed in the present study clustered into a highly supported subclade with *T. glossogobae* (ON042773; Qin et al. 2023), *T. ruditapicis* (FJ499385; Zhan et al. 2009), and *T. sinonovaculae* (FJ499386; Zhan et al. 2009). The subclade is part of the clade comprising the majority *Trichodina* species, as well as sequences attributed to the genera *Trichodinella* (HQ407386–87, JQ663869, MW311496–99, and ON042780) and *Tripartiella* (MT777618 and MT777619). Since the latter sequences are embedded within those of the genus *Trichodina*, their generic classification is likely incorrect.

**FIGURE 3:**
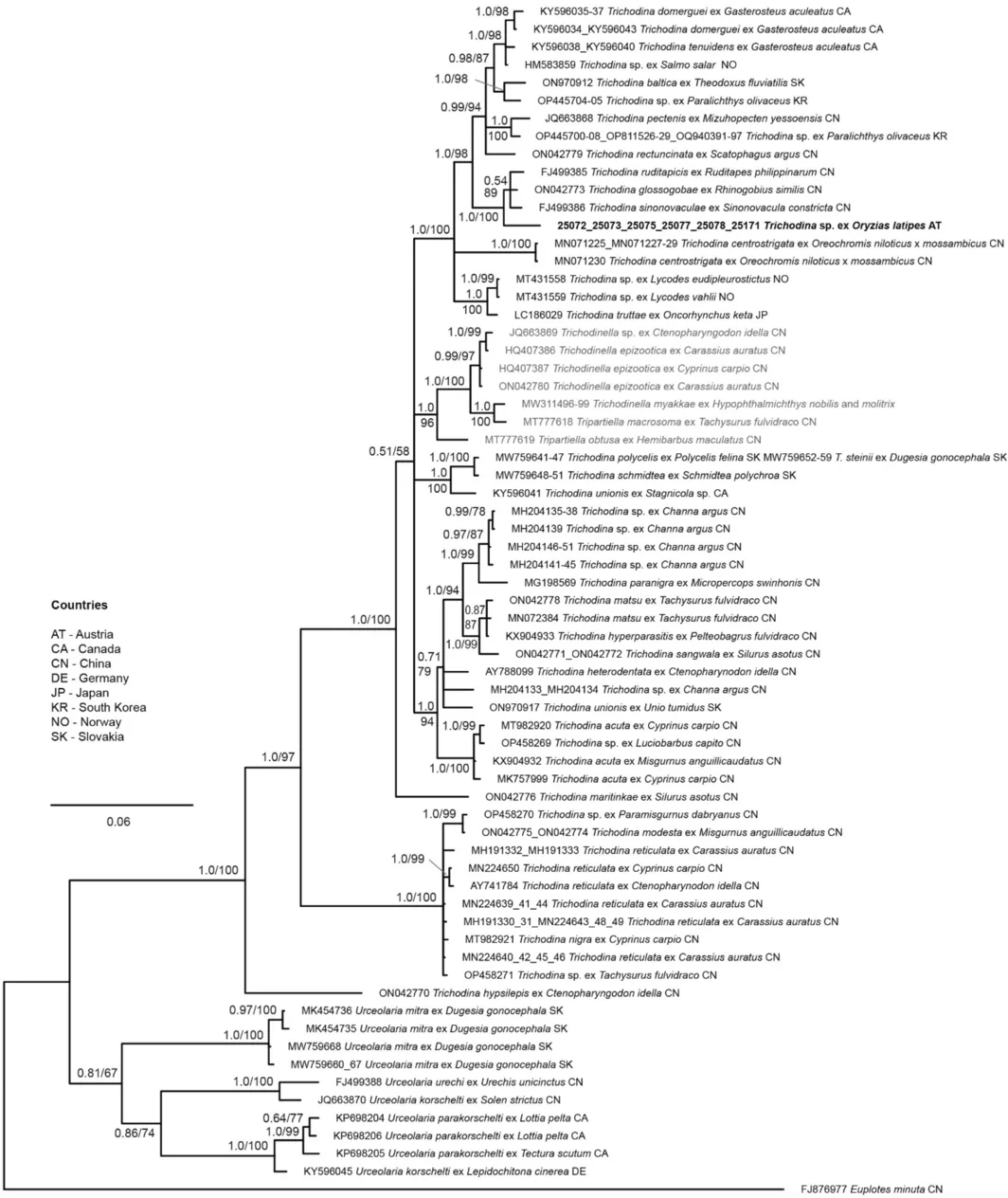
Bayesian Inference tree based on partial (1160 bp) 18S rRNA gene sequences of ciliates belonging to the order Mobilida. The names of the host species and countries are provided for each record. Bayesian posterior probabilities and Maximum Likelihood bootstrap support values are provided at all nodes. The scale bar indicates the expected mean number of substitutions per site according to the model of sequence evolution applied.

The histological examination was carried out on 4 μm serial cuts of one fish and on small organ pieces from two other specimens. The samples were immediately fixed in 4% buffered formalin and processed following routine histopathology protocols, with a final H&E staining of the slides. *Trichodina* parasites were visible in collecting tubes of the kidney (Figure 4), and multifocal granulomatous and necrotic lesions were present in the spleen and liver. Since the *Trichodina* parasites were detected in wet mounts of kidney samples of only two individual fish and the samples were preserved for molecular and histological examinations, the available material for the morphological description was limited to photographs of the parasites embedded in tissue (Figure 2). Preliminary morphometric measurements were taken according to Lom (1958), but it was not possible to assess the morphometrics of the denticles. The collected data are summarized in Table 2.

**FIGURE 4:**
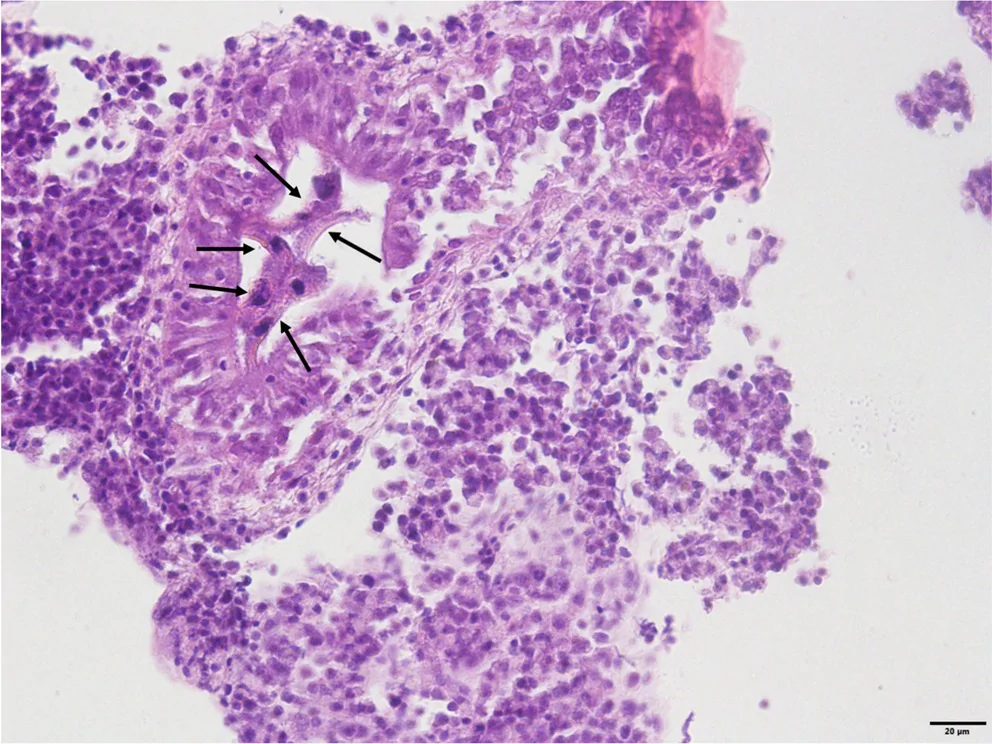
Fragments of five *Trichodina* sp. in the lumen of a collecting tubule of the kidney. H&E stain.

**TABLE 2:** Morphological comparison of *Trichodina* sp. from this study with the genetically closets species based on available sequence data from NCBI GenBank.

	*Trichodina glossogobae*	*Trichodina ruditapicis*	*Trichodina sinonovaculae*	This study
Host	*Glossogobius giuris*	*Ruditapes philippinarum*	*Sinonovacula constricta*	*Oryzias latipes*
Diameter of the body	30.6–46.9	48–71	61–78	35*–*44.6
Diameter of the adhesive disc	24.5–38.7	34–48	48–67	32.5–38
Border membrane	2.6–4.6	4–5.5	5–8	3.5*–*4.0
Denticular ring diameter	15.3–28.6	22–28	25–36	23.3–25.0
Number of radial denticles	22–24	22–26	24–27	22*–*25
Number of radial pins	6–9	7–10	9–10	7*–*8
Tissue location	Gills	Gills	Gills	Collecting tubes of kidney
GenBank accession no. of this study	ON042773 (Qin et al. 2023)	FJ499385 (Zhan et al. 2009)	FJ499386 (Zhan et al. 2009)	Under submission
Reference (morphological data)	Mitra et al. 2011	Xu et al. 2000	Xu et al. 1999	

## 3 Discussion

The finding of *Trichodina* sp. in the head kidney of fish seems to be a rather rare event. Notably, in medakas, both nephrons and interstitial lymphomyeloid tissues can be found in the head kidney and in the trunk kidney, and the head kidney is much larger than the trunk kidney (Fedorova et al. 2008). This contrasts with most teleost species, where nephrons are not present in the head kidney. The genuine anatomical feature of the medaka kidney enabled the parasites to migrate to the most cranial parts of the organ. Still, histology did not detect an obvious inflammatory reaction or other pathologies caused by the ciliates. Nevertheless, this observation should be viewed with caution, since the sample number was too low for a reliable conclusion on the effects of the infestation. Moreover, several other pathogens were present in the stock, first and foremost a severe infection with *Mycobacterium* sp. The co‐infection may have contributed to the massive spread of the endozoic trichodinid parasites but does not explain their presence.

A thorough morphological description of the parasite was not possible due to the small sample number and the use of the samples for different examination methods. The measurements were based on photos and compared to the genetically closest *Trichodina* spp. and species described from the urogenital tract of fishes (Tables 1, 2). Among the three genetically closely related species, morphological similarities were observed in *T. glossogobae* but not in the mussel parasites *T. ruditapicis* and *T. sinonovaculae*. A morphological comparison to trichodinid species parasitizing the urogenital tract of fish showed the closest similarity to *Paratrichodina alburni* Lom 1963 and *T. schizothoraci* Aschurova & Stein 1972, respectively. However, the species detected in the present study could not be assigned to any of them due to a lack of morphological data and the absence of corresponding sequence data. The high prevalence of the endozoic *Trichodina* sp. in the absence of ectoparasitic trichodinids suggests a well‐established tissue‐specific species which certainly has the potential to impact renal function and thus the health status of the stock. This is of special interest, since medakas are frequently used in research facilities for various purposes, and unknown infections may influence research results. Most such facilities carry out regular health screenings for the detection of targeted pathogens, mostly based on molecular methods. Our finding outlines the importance of including histopathology to routine monitoring in fish research facilities to not miss unexpected pathogens. Moreover, we present a reliable molecular method for the detection of *Trichodina* spp. and the first sequence data of an endozoic trichodinid from fish.

## Author Contributions

**E. Lewisch:** conceptualization, investigation, methodology, data curation, supervision, writing – original draft, project administration. **S. Thalguter:** methodology, writing – review and editing. **K. Lipnik:** writing – review and editing, methodology. **J. Harl:** methodology, writing – review and editing, formal analysis.

## Funding

The authors have nothing to report.

## Conflicts of Interest

The authors declare no conflicts of interest.

## Data Availability

The data supporting the findings of this study are available within the article. Any data not included in the manuscript will be made available upon reasonable request.
